# Comparison of the Anti-Inflammatory Activities of Supercritical Carbon Dioxide versus Ethanol Extracts from Leaves of *Perilla frutescens* Britt. Radiation Mutant

**DOI:** 10.3390/molecules22020311

**Published:** 2017-02-17

**Authors:** Chang Hyun Jin, Han Chul Park, Yangkang So, Bomi Nam, Sung Nim Han, Jin-Baek Kim

**Affiliations:** 1 Advanced Radiation Technology Institute, Korea Atomic Energy Research Institute, Jeongeup-si, Jeollabuk-do 56212, Korea; chjin@kaeri.re.kr (C.H.J.); phc3634@kaeri.re.kr (H.C.P.); yangkang@kaeri.re.kr (Y.S.); bomi1201@kaeri.re.kr (B.N.); 2 Department of Food and Nutrition, College of Human Ecology, Seoul National University, 1 Gwanak-ro, Gwanak-gu, Seoul 08826, Korea; snhan@snu.ac.kr

**Keywords:** radiation mutant, *Perilla frutescens*, extraction, supercritical carbon dioxide, ethanol, isoegomaketone

## Abstract

In this study, we aimed to compare supercritical carbon dioxide extraction and ethanol extraction for isoegomaketone (IK) content in perilla leaf extracts and to identify the optimal method. We measured the IK concentration using HPLC and inflammatory mediators in lipopolysaccharide (LPS)-stimulated RAW 264.7 cells from the extracts. The IK concentration was 10-fold higher in perilla leaf extracts by supercritical carbon dioxide extraction (SFE) compared with that in perilla leaf extracts by ethanol extraction (EE). When the extracts were treated in LPS-induced RAW 264.7 cells at 25 µg/mL, the SFE inhibited the expression of inflammatory mediators such as nitric oxide (NO), monocyte chemoattractant protein-1 (MCP-1), interleutkin-6 (IL-6), interferon-β (IFN-β), and inducible nitric oxide synthase (iNOS) to a much greater extent compared with EE. Taken together, supercritical carbon dioxide extraction is considered the optimal process for obtaining high IK content and anti-inflammatory activities in leaf extracts from the *P. frutescens* Britt. radiation mutant.

## 1. Introduction

*Perilla frutescens* (L.) Britt. is an annual herbaceous plant in the Lamiaceae family, which has been widely cultivated in India, China, Japan, and Korea. Its leaves are used in Asian cuisines, and its seeds are used to for the extraction of edible oil in Korea. It is also commonly used in traditional Chinese medicine. *P. frutescens* contains several components including rosmarinic acid, luteolin, apigenin, ferulic acid, (+)-catechin, triterpenoids, and caffeic acid [1,2]. Recent studies demonstrated the pharmacological activities of extracts from *P. frutescens*. Ethanol extracts from *P. frutesens* leaves were shown to possess anti-cancer [3], anti-inflammatory [4], and anti-bacterial [5] activities. In addition, methanol extracts from *P. frutescens* leaves showed anti-allergy, anti-inflammatory [6], and anti-cancer [7] activities. Water extracts from *P. frutescens* leaves improved gastrointestinal discomfort [8] and suppressed tumor necrosis factor-alpha production in mice [9].

Previously, we identified a radiation mutant *P. frutescens* Britt. with an isoegomaketone (IK) level approximately 10-fold greater than that of the wild-type [10]. IK, an essential oil component in *P. frutescens*, exhibits several biological activities. It has been shown to suppress NO production in LPS-treated RAW264.7 cells [11] and to induce apoptosis in several cancer cells through both caspase-dependent and caspase-independent pathways [12,13].

Supercritical carbon dioxide (SC-CO_2_) extraction is a novel and powerful technique for extracting lipophilic components [14,15]. SC-CO_2_ extraction has several advantages over the use of organic solvents, because CO_2_ is non-toxic, non-reactive, non-corrosive, and inexpensive. SC-CO_2_ extraction of *P. frutescens* seeds has been performed previously [16,17], but the SC-CO_2_ extraction method has not been used for the extraction of IK from *P. frutescens* leaves. In the present study, we investigated the optimal extraction method for the leaves of the *P. frutescens* radiation mutant for IK contents and anti-inflammatory activities comparing SFE with EE.

## 2. Results and Discussion

### 2.1. Yield and Composition of SFE and EE

Previously, we identified a mutant *P. frutescens* Britt. obtained by mutagenesis using gamma rays [11], which had much higher anti-inflammatory activity than that of the wild-type control. After HPLC analysis and assay-based purification of the mutant, we showed that the enhanced anti-inflammatory activity was due to a 10-fold increase in IK content in the leaves compared with the wild-type [10]. There have been many reports regarding extraction from perilla using organic solvents [3,4,5,6,7,8,9], SC-CO_2_ [16,17], and microwave-assisted techniques [18]. In this study, we focused on the SC-CO_2_ method, because the mutant *P. frutescens* has a high content of IK, a kind of oil component, and the SC-CO_2_ method had never been used for obtaining extract from perilla leaves. Perilla oil is present mostly in the seeds; therefore, the SC-CO_2_ technique was used previously only on seeds and not leaves. However, the IK content was approximately five-fold higher in leaves compared with seeds from the mutant *P. frutescens* (data not shown). Generally, the water extraction method is used in the food industries, because it is an efficient and environmentally friendly technique for extracting various compounds from plants [19]. In addition, it has the advantage of possibilities for various forms of food processing. However, it was not a good method for extracting IK from perilla leaves. The extract from the water extraction method from mutant perilla leaves did not contain IK content. The extraction of IK from mutant perilla leaves had been accomplished by employing organic solvents such as methanol, ethanol, or hexane. However, in the case of using organic solvents for extraction, an additional process to evaporate these solvents from extracts is required. In addition, there is increasing public concern regarding the possibility of toxic solvent residues remaining in the final product. For the above reasons, we used the SC-CO_2_ method to extract IK from the mutant perilla leaves.

**Figure 1:**
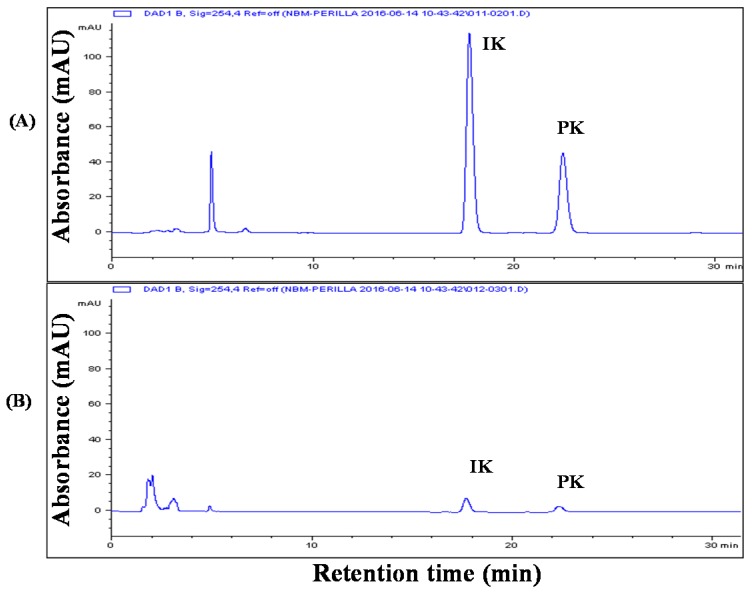
HPLC chromatograms of (**A**) SFE (50 °C, 400 bar, 3 h, and CO_2_ flow rate of 60 mL/min) and (**B**) EE (shaking incubation for 6 h at 60 °C) at 254 nm.

In this study, we obtained extracts from *P. frutescens* radiation mutant leaves using supercritical carbon dioxide extraction and ethanol extraction. To our knowledge, this is the first study to apply the SC-CO_2_ extraction technique to perilla leaves. Generally, temperature and pressure have an influence on the SC-CO_2_ extraction yield. In the case of perilla seeds, when the pressure was above 340 bar, the yield was saturated with 3 kg CO_2_ regardless of the temperature and pressure [17]. In addition, the solubility of perilla oil at 400 bar in SC-CO_2_ was constant at all temperatures [17]. To obtain the maximum SC-CO_2_ extraction yield from perilla leaves, we used sufficient CO_2_ at 400 bar. The extraction yields of SFE and EE were 5.0% ± 0.2% and 9.0% ± 0.2%, respectively. Figure 1 shows the compositions of the two extracts. SFE contained three main oil components, including isoegomaketone (IK) and perilla ketone (PK), but EE contained numerous components, including polar and nonpolar substances. These results were caused by the difference in solubility between ethanol and CO_2_. IK and PK contents were approximately 10-fold higher in SFE compared with EE. The IK content was 6.3 ± 0.2 mg/g and 63.8 ± 2.6 mg/g in EE and SFE, respectively. The PK content was 13.3 ± 0.3 mg/g and 146.9 ± 5.6 mg/g in EE and SFE, respectively. While the extraction yield from the SC-CO_2_ extraction method was lower than that from the ethanol extraction method, SC-CO_2_ extraction was more effective in obtaining an extract with a higher IK content from *P. frutescens* radiation mutant leaves.

### 2.2. Effect of SFE and EE on Cell Viability

**Figure 2:**
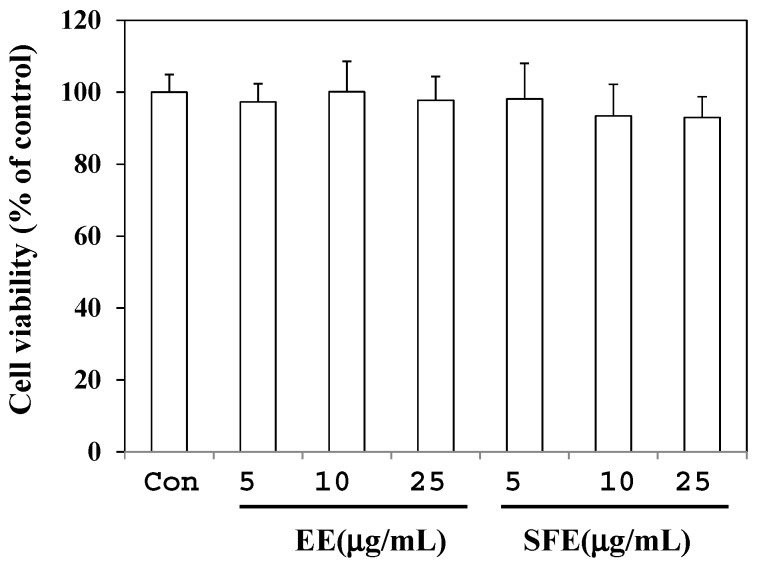
Effects of SFE (50 °C, 400 bar, 3 h, and CO_2_ flow rate of 60 mL/min) and EE (shaking incubation for 6 h at 60 °C) on cell viability. Cell viability was determined using the EZ-Cytox cell viability assay kit. The cells were treated with various concentrations of SFE and EE for 24 h. After the incubation period, 10 μL of the kit solution were added to each well and incubated for an additional 4 h. Data are presented as means ± SD (*n* = 3). *p* < 0.05 vs. control.

To measure the cytotoxicity of SFE and EE, RAW 264.7 cells were treated with two extracts at concentrations of 5, 10, and 25 µg/mL for 24 h. As shown in Figure 2, neither extract affected cell viability at the concentrations lower than 25 µg/mL. Therefore, we performed all the experiments using treatment with extracts lower than 25 µg/mL.

### 2.3. Effects of SFE and EE on LPS-Stimulated NO Production in RAW 264.7 Cells

**Figure 3:**
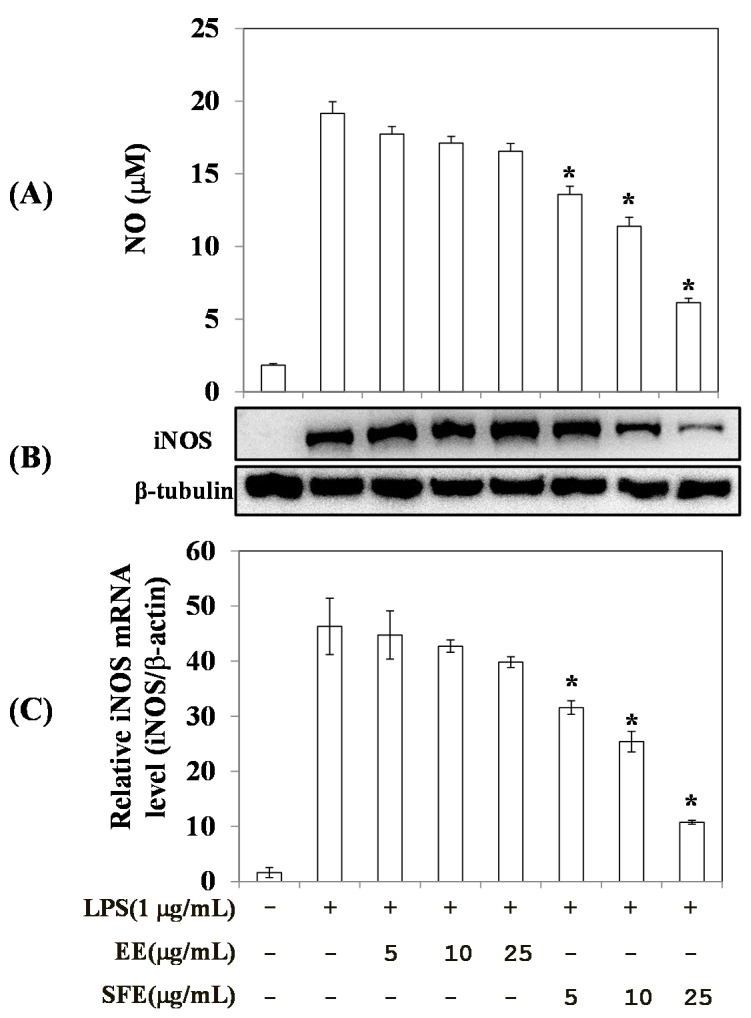
Effects of SFE (50 °C, 400 bar, 3 h, and CO_2_ flow rate of 60 mL/min) and EE (shaking incubation for 6 h at 60 °C) on NO production and iNOS expression levels in RAW 264.7 cells. (**A**) Cellular media (100 µL) were mixed with equal volumes of Griess reagent. Nitrite levels were measured as an indicator of NO production as described in the Materials and Methods section. Data are presented as means ± SD (*n* = 4). * *p* < 0.05 vs. the LPS-alone group; (**B**) Whole-cell lysates were prepared, in which the expression level of iNOS protein was measured by Western blot analysis. The results were confirmed by two independent experiments; (**C**) Total RNA was isolated and used to measure the expression level of iNOS mRNA by quantitative real-time PCR. Data are presented as means ± SD (*n* = 3). * *p* < 0.05 vs. LPS alone−treated group.

We first compared the anti-inflammatory effects of SFE and EE on nitric oxide (NO) production in LPS-treated RAW 264.7 cells. NO is a potentially toxic gas produced from the amino acid l-arginine via nitric oxide synthase (NOS) activity. Appropriate levels of NO are important for organ protection, but excessive NO production is associated with many diseases, including carcinogenesis, arthritis, and diabetes [20,21]. RAW 264.7 cells were treated with SFE and EE for 2 h before stimulation with 1 µg/mL LPS for 18 h. NO production increased significantly after incubation with LPS (Figure 3A). Both extracts decreased the levels of LPS-stimulated NO production in a dose-dependent manner. However, SFE exerted five-fold greater inhibitory activity on NO production compared with EE (Figure 3A). To determine whether the suppression of NO production by SFE and EE was due to the inhibition of iNOS expression, we measured protein and mRNA levels of iNOS. Western blot analyses showed that LPS-induced increases in iNOS levels were attenuated by treatment with SFE in a dose-dependent manner (Figure 3B). Furthermore, RT-PCR analyses showed that the iNOS mRNA level was increased by LPS stimulation and this increase was significantly reduced by SFE treatment in a dose-dependent manner (Figure 3C). While EE also decreased the iNOS mRNA level, its magnitude of inhibition was much lower than that of SFE. These results indicate that IK plays an important role in the anti-inflammatory activity of SFE and has greater anti-inflammatory activity than the polar components in EE.

### 2.4. Effects of SFE and EE on Production of Inflammatory Mediators in LPS-Stimulated RAW 264.7 Cells

**Figure 4:**
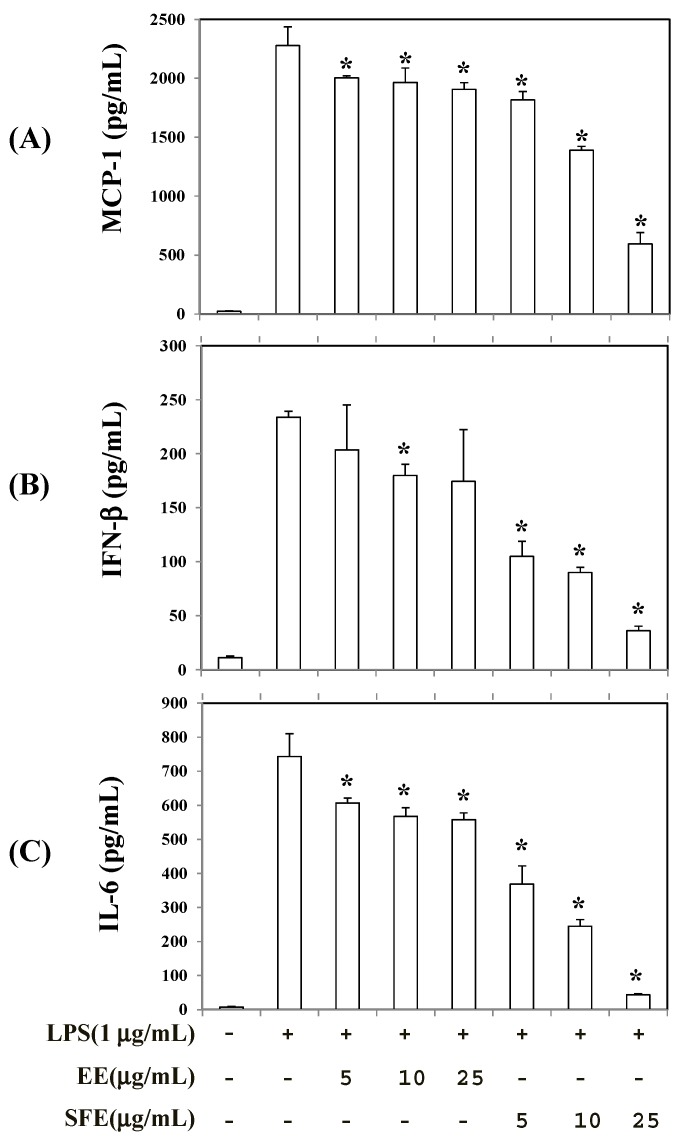
Effects of SFE (50 °C, 400 bar, 3 h, and CO_2_ flow rate of 60 mL/min) and EE (shaking incubation for 6 h at 60 °C) on the production of inflammatory mediators in RAW 264.7 cells. RAW 264.7 cells were treated with each extract for 2 h prior to addition of LPS (1 µg/mL) and further incubated for 4 h. MCP-1 (**A**); IFN-β (**B**); and IL-6 (**C**) levels were measured in the cellular medium using an ELISA kit. Data are presented as means ± SD (*n* = 3). * *p* < 0.05 vs. LPS alone−treated group.

**Figure 5:**
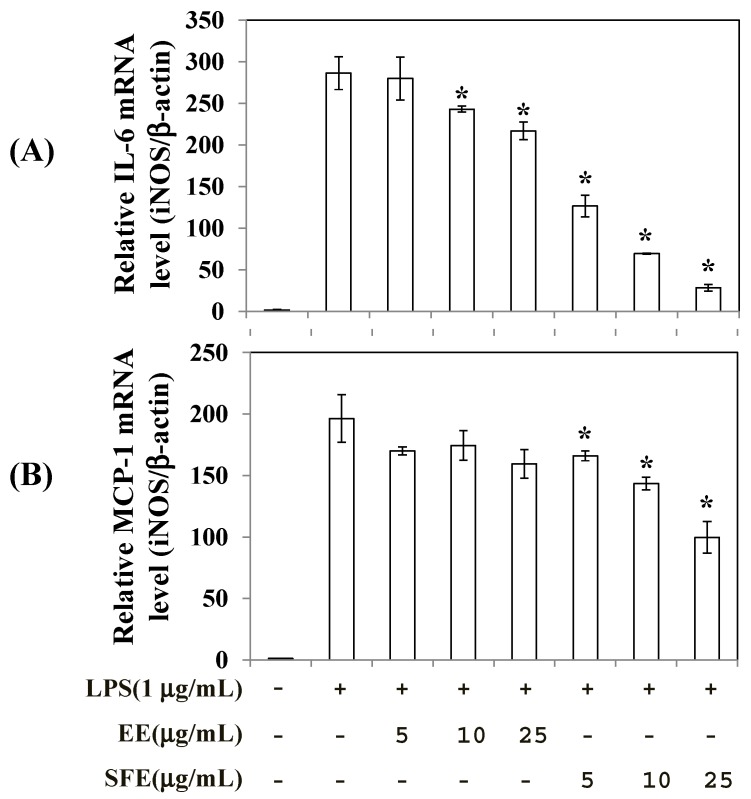
Effects of SFE (50 °C, 400 bar, 3 h, and CO_2_ flow rate of 60 mL/min) and EE (shaking incubation for 6 h at 60 °C) on IL-6 and MCP-1 expression levels in RAW 264.7 cells. Total RNA was isolated and used to measure the expression levels of IL-6 (**A**) and MCP-1 (**B**) mRNA by quantitative real-time PCR. Data are presented as means ± SD (*n* = 3). * *p* < 0.05 vs. LPS alone−treated group.

To determine the effects of SFE and EE treatment on the production of inflammatory mediators, RAW 264.7 cells were treated with SFE and EE for 2 h before stimulation with 1 µg/mL LPS for 4 h and the levels of monocyte chemoattractant protein-1 (MCP-1), interferon-β (IFN-β), and interleukin-6 (IL-6) were measured. As shown in Figure 4, both SFE and EE treatments suppressed the production of MCP-1, IFN-β, and IL-6 in LPS-stimulated RAW 264.7 cells. However, SFE showed about three- to four-fold stronger inhibitory activity on the production of all inflammatory mediators compared with EE. Furthermore, SFE treatment lowered IL-6 and MCP-1 mRNA levels to a greater extent than EE (Figure 5).

IK, an essential oil present in *P. frutescens*, exhibits several biological activities, including anti-inflammatory [11] and anti-cancer effects [12,13]. While SFE had a higher IK content, EE contained several anti-inflammatory polar compounds, such as pomolic acid, tormentic acid, corosolic acid [22], and rosmarinic acid methyl ester [23]. However, the concentration of these compounds in EE was too low to show anti-inflammatory activities in LPS-stimulated RAW 264.7 cells. The superior anti-inflammatory activities of SFE resulted from the higher IK content. Furthermore, we tried to adopt SFE and EE into food processing. Unlike SFE, EE had a disadvantage due to the mixture of both polar and nonpolar ingredients. Therefore, SC-CO_2_ is a much more effective method of acquiring extract from mutant perilla leaves for the future development of functional foods.

## 3. Materials and Methods

### 3.1. Materials

Leaves of *P. frutescens* Britt. radiation mutant were harvested at Advanced Radiation Technology Institute (Jeongeup, Korea). DMEM and fetal bovine serum (FBS) were purchased from Hyclone (Logan, UT, USA). LPS, phenylmethylsulfonyl fluoride, sodium nitrite, DMSO, Griess reagent, and protease inhibitor cocktail were purchased from Sigma-Aldrich (St. Louis, MO, USA). Goat anti-rabbit IgG HRP-conjugated antibody was purchased from Invitrogen (Carlsbad, CA, USA). The RNeasy kit was purchased from QIAGEN (Valencia, CA, USA). The EZ-Cytox Cell Viability assay kit was purchased from Daeil Lab Services (Seoul, Korea). The Advantage RT-for-PCR kit was purchased from Clontech (Mountain View, CA, USA). SYBR Premix was purchased from Takara Bio Inc (Shiga, Japan). NP40 cell lysis buffer was purchased from Biosource (San Jose, CA, USA). Rabbit polyclonal antibodies against β-tubulin and iNOS were purchased from Santa Cruz Biotechnology (Santa Cruz, CA, USA).

### 3.2. Cell Culture

RAW 264.7 cells were cultured in DMEM supplemented with 10% FBS, penicillin (100 U/mL), and streptomycin (100 μg/mL) and incubated at 37 °C in an atmosphere of 5% CO_2_. 

### 3.3. Ethanol Extraction

The dried leaves of *P. frutescens* (10 g) were extracted with ethanol (100 mL) in a shaking incubator for 6 h at 60 °C and filtered through filter paper (Whatman No. 4). Ethanol was of analytical grade (≥95.0%) and obtained from Duksan Co. (Seoul, Korea). The moisture content in dried sample was found to be 5.3% ± 1.4%. The solvent was evaporated in vacuo to afford the ethanol extract (0.9 g). Ethanol extraction was repeated three times.

### 3.4. SC-CO_2_ Extraction

A laboratory-scale supercritical fluid extraction system (Ilshin Autoclave Co., Daejeon, Korea) was used for SC-CO_2_ extraction of perilla leaves. The dried perilla leaves were ground using a milling machine, and the powder (180 g) was transferred to an extraction column. The moisture content in the powder sample was found to be 5.3% ± 1.4%. The powder sample was held in place within the extraction column by glass wool mounted on both ends of the extractor. After the extractor reached the predetermined temperature (50 °C) and pressure (400 bar), the sample was allowed to stand for 10 min for temperature (50 °C) and pressure (400 bar) equilibration. Then, the extraction was performed by passing the CO_2_ (99.9%) through the column at a flow rate of 60 mL/min at 50 °C and 400 bar for 3 h. The extracted oil was separated by pressure reduction and collected in the trap. The collected oils were stored in a refrigerator at 4 °C. SC-CO_2_ extraction was repeated two times.

### 3.5. HPLC Analysis

HPLC analysis was conducted using the Agilent Technologies model 1100 instrument (Agilent Technologies, Santa Clara, CA, USA). The samples were analyzed by reverse phase (C18) HPLC analysis (YMC-Triart C18, 4.6 × 250 mm I.D, S-5 μm, flow rate 1 mL/min, UV detection: 254 nm) using acetonitrile:water (44:55 to 55:45, 30 min) as the gradient solvent. Solvents used in HPLC analysis were of analytical grade (≥99.9%) and obtained from Sigma Chemical Co. (St. Louis, MO, USA).

### 3.6. Cytotoxicity Assay

To measure cell viability, we used the EZ-Cytox cell viability assay kit (Daeil Lab Service). The cells were cultured in a 96-well flat-bottom plate at a density of 2.0 × 10^5^ cells/mL for 24 h. The cells were subsequently treated with various concentrations of the extracts for an additional 24 h. After the incubation period, 10 μL EZ-Cytox were added to each well and incubated for 4 h at 37 °C and 5% CO_2_. Cell viability was determined by measuring formazan production using an ELISA reader at an absorbance of 480 nm with a reference wavelength of 650 nm.

### 3.7. Determination of NO Concentration

Nitrite in the cellular media was measured by the Griess method [24]. The cells were cultured in a 96-well plate and treated with LPS (1 μg/mL) for 18 h. The medium was collected at the end of the culture period for determination of nitrite production. Equal volumes of Griess reagent and cellular supernatant were mixed, and the absorbance was measured at 540 nm. The concentration of nitrite (μM) was calculated using a standard curve generated from known concentrations of sodium nitrite dissolved in DMEM. The results are presented as the means ± SD of four replicates in one representative experiment.

### 3.8. Preparation of Cell Extracts and Western Blot Analysis

Cells were washed once with cold PBS and harvested by pipetting. For whole-cell extract preparation, the cells were lysed in NP40-based cell lysis buffer containing protease inhibitor cocktail (Sigma, St. Louis, MO, USA) and phenylmethylsulfonyl fluoride (Sigma) for 30 min on ice. The protein concentration of the cell lysate was determined using the Bio-Rad Protein Assay (Bio-Rad, Hercules, CA, USA). Aliquots of 50 µg protein were loaded and electrophoresed on 10% SDS-polyacrylamide gels and then transferred to nitrocellulose membranes (Hybond ECL Nitrocellulose; GE Healthcare, Chandler, AZ, USA). The membranes were washed once with wash buffer consisting of PBS with 0.05% Tween 20 and blocked with blocking buffer consisting of PBS with 5% skim milk and 0.05% Tween 20 for 1 h. After blocking, the membranes were incubated with rabbit anti-HO-1 or anti-β-tubulin primary antibody overnight at 4 °C. Rabbit anti-iNOS polyclonal antibody was diluted 1:1000, and rabbit anti-β-tubulin polyclonal antibody was diluted 1:200 in blocking buffer. After incubation, the membranes were washed and subsequently incubated for 1 h at room temperature with goat anti-rabbit IgG HRP-conjugated secondary antibody diluted 1:5000 in blocking buffer. The membranes were washed and the protein bands detected by chemiluminescence analysis (GE Healthcare).

### 3.9. Quantitative Real-Time PCR

The cells (2 × 10^5^ cell/mL) were cultured in a 100 mm petri dish for 24 h. Total RNA was isolated using the RNeasy Kit according to the manufacturer’s instructions. The Advantage RT-for-PCR kit was used for reverse transcription according to the manufacturer’s protocol. The Chromo4 real-time PCR detection system (Bio-Rad) and iTaqTM SYBRR Green Supermix (Bio-Rad) were used for RT-PCR amplification of HO-1 and β-actin under the following conditions: 50 cycles of 94 °C for 20 s, 60 °C for 20 s and 72 °C for 30 s. All of the reactions were repeated independently at least three times to ensure reproducibility of the results. Primers were purchased from Bioneer Corp (Daejeon, Korea). Primer sequences are shown in Table 1.

### 3.10. Measurement of MCP-1, IFN-β, and IL-6 by ELISA

The quantities of MCP-1, IFN-β, and IL-6 in the culture medium were measured using an ELISA kit (R&D Systems, Minneapolis, MN, USA) according to the manufacturer’s protocol. The results are presented as the means ± SD of three replicates from one representative experiment.

### 3.11. Statistical Methods

All data are presented as means ± standard deviation (SD). The differences between the means of the treated and untreated groups were determined with Student’s *t* tests implemented in the Excel program (Microsoft Corp., Redmond, WA, USA); *p* values < 0.05 were considered to indicate statistical significance.

## 4. Conclusions

In this study, we obtained extracts from mutant perilla leaves using the SC-CO_2_ method and the ethanol extraction method. SFE exhibited an approximately 10-fold higher IK content and much stronger anti-inflammatory activity compared with EE. SFE has also the advantage for applications in food processing. Therefore, SFE is a much more effective material for the future development of functional foods.

## Figures and Tables

**Table 1:** Primers sequences for real-time PCR analysis.

Target Gene	5′ to 3′ Direction	
iNOS	Forward	TGAGAGGGAAATCGTGCGTGAC
	Revers	GCTCGTTGCCAATAGTGATGACC
IL-6	Forward	GTTCTCTGGGAAATCGTGGAA
	Revers	GCAAGTGCATCATCGTTGTTC
MCP-1	Forward	GCATCTGCCCTAAGGTCTTCA
	Revers	AAGTGCTTGAGGTGGTTGTGG
β-actin	Forward	TCCTACACCACACCAAACTGTGTGC
	Revers	CTCCAATCTCTGCCTATCCGTCTC
